# Clinic of Jefferson Medical College

**Published:** 1844-11-30

**Authors:** 


### THE MEDICAL EXAMINER,

### And Record of Medical Science.

Vol. VII.] PHILADELPHIA, SATURDAY, NOVEMBER 30, 1844. [No. 24.

### CLINICAL LECTURES AND REPORTS.

### PHILADELPHIA HOSPITAL.

CLINIC OF JEFFERSON MEDICAL COLLEGE.

*November* 16, 1844.

BY PROFESSOR DUNGLISON.

(Reported by Mr. Samuel G. White.)

Professor Dunglison repeated his remark, made at the conclusion of the last clinical lecture, that he should endeavour to present to the class, for clinical observation, such cases as would tend to elucidate the various points to which their attention had been directed, during the week, from the chair of Institutes in the College.

In the present instance, however, he deemed it advisable to deviate somewhat from this plan, by commencing the lecture with some remarks on a well marked case of the affection recently distinguished as

TYPHOID FEVER, of the existence of which, in the wards, he was not aware until his arrival this morning at the Hospital.

The chain of morbid phenomena constituting this disease, it is well known, many have referred to a peculiar intestinal lesion, in which the mucous follicles—glands of Peyer—near the termination of the small intestines, form a prominent feature.

The lecturer, from his own observations, and a careful examination of the recorded observations of others, is not disposed to coincide in opinion with those who consider typhoid fever to be merely symptomatic of this lesion, and a separate and distinct disease from typhus; but he is rather induced to believe that they, with the black-tongue fever, are different varieties of adynamic fever.

As to the true nature of the disease, various opinions are entertained by different observers. Some, with Bretonneau, consider the essence of the affection to consist in the intestinal lesion, and hence the term dothinenteritis applied to it. Others view the lesion, with the *taches rouges*, in the same light with the eruption of variola, morbilli, scarlatina, &c., as merely expressions of the disease, and not absolutely necessary to its existence.

From extensive observations made in England, and elsewhere, both in public and private practice, it would seem to be probable that different phenomena may occur as expressions of the same adynamic fever; that the same symptoms may be present with the intestinal lesion, constituting typhoid, or without it as in typhus, and the nature of the affection may only be determined, in many instances at least, by a *post mortem* examination. How are we, then, to determine, when summoned to a patient, which affection is present? Is the judgment in those cases to be suspended until a necroscopical investigation reveals the only feature of distinction, the intestional lesion? This must certainly be requisite in some instances, for the two diseases often so closely resemble each other as to be distinguishable in no other manner.

According to the severity of symptoms, adynamic fever was divided by the older writers into typhus gravior, and typhus mitior—the former corresponding with the typhus of modern writers; while the latter included the typhoid fever as described by certain recent authors.

Typhus, admitting the modern distinctions, is a disease very prevalent in England, whilst it is extremely rare both in Paris and this country; but the reverse is true in respect to typhoid fever. To this circumstance may be attributed the discrepancies in the description of continued fever, which exist among English and French writers—the former contending that the intestinal lesion is seldom,—the latter that it is always, present; the English considering it accidental, whilst many of the French believe it to be the pathological character of their fever,—both accurately describing the febrile variety, which they are accustomed respectively to meet with.

Some admit only this distinction in continued fever, that where there is no intestinal disease, it is typhus; where there is, it is typhoid; but the bent of the lecturer's observations is towards the belief, that both are different expressions of adynamic fever, for in the same epidemic identical vital phenomena may be present in cases with and without the intestinal lesion.

As usually described, the vital manifestations of typhoid fever are well marked—there is headache, or tension across the forehead—epistaxis, confusion of intellect, peculiar expression of countenance, somnolency, more or less delirium and stupor, diarrhœa, meteorism, gurgling, with pain on pressure in the right iliac fossa, &c. About the close of the first week, a peculiar eruption, the *taches rouges*, may be discovered by careful examination in the majority of cases.

It commonly appears, first about the chest and abdomen, in the form of rose colored lenticular spots, slightly elevated above the surface, which are almost entirely dissipated by passing the finger over them; the redness immediately returning after the finger has been removed.

[These *taches rouges* were exhibited by the Professor to the class, on the patient before him, and were distinctly seen.]

Besides these, which are considered peculiar to typhoid fever,—at a later period may be seen a number of minute vesicles—sudamina—scattered over the abdomen and other parts of the surface. They are not peculiar to the disease, though it is stated they occur more frequently in it than any other, but seem to be closely connected with the cutaneous exhalation; in fact they bear some ratio to the amount of perspiration, and are most abundant in those parts where the sweat naturally collects, as the axilla, inguinal region, &c. The expression of countenance in typhoid is very peculiar, so much so as to be easily recognized after it has once been seen. In the present instance the patient—a female—was admitted for ordinary fever, but in passing the wards immediately before lecture, the lecturer was struck with her appearance, and examination proved it to be a case of this disease.

The patient now under consideration exhibits several of the phenomena, just alluded to, in a very marked degree. The peculiar expression of countenance, mentioned as so characteristic, is very obvious. The *taches rouges* are unusually numerous, the chest and abdomen being very thickly studded with them. Pressure on the right iliac fossa elicits the gurgling, but produces no pain. There is some diarrhœa, and there has been epistaxis, with headache and giddiness. No sudamina are as yet perceptible, although they may occur at a later period. Inasmuch as they are so generally present, there seems no impropriety in considering the *taches rouges* to be as much a characteristic expression of typhoid fever, as the ulceration of the intestinal follicles. In the present case, there is not much, if any, developement of the spleen, which may often be felt below the margin of the ribs.

The treatment of fever in general has very much changed in recent periods. Formerly, it was customary to bleed freely and purge violently in every case, but that system has been generally replaced by one more philosophical, suggested by "rational therapeutics." It is now very simple. Instead of giving enormous doses of calomel and jalap, and other cathartics, which would call the patient frequently out of bed—ten or twelve times in the twenty-four hours—and thus counteract any good that might arise from quietude in other respects, we adopt a very different course. Violent purgatives, by augmenting the peristaltic action of the intestines to an inordinate degree, cannot but increase any existing irritation of the digestive apparatus. We now know, that the most soothing measures are the most fraught with benefit in all febrile disturbances, and *à fortiori* in typhoid fever, where there is ulceration of the intestinal follicles. The mildest means are, therefore, adopted—the bowels are kept open and the morbid secretions removed by gentle cathartics, as castor oil, or by enemata; the tongue, which is usually dry and cracked, is moistened by the use of iced water, and the surface kept in a temperate condition by cool applications, such as sponging with cold water, &c.

Later in the disease, when the strength begins to fail, gentle excitants, as tonics, may be administered, but caution is required to avoid over-stimulation, which could not fail to be injurious.

As these fevers cannot be cut short, the main care of the physician should be to rectify deranging circumstances and to support the system until the malignant influence has passed away. Occasionally, benefit may be derived from making counter-irritation over the iliac fossa, or by covering the same region with poultices or emollient applications. The disease has a natural tendency to run a fixed course, a circumstance which should not be lost sight of in the treatment. By adopting the mode of management indicated above, we may succeed in conducting cases to a favorable termination, when by more violent plans a fatal issue might have been the consequence.

### DISEASES OF IMBIBITION.

The Professor then proceeded to make a few observations on several pathological cases illustrative of the property of penetrability possessed by animal membrane, a subject, which had occupied the consideration for the class, during several days past, at the Jefferson Medical College. It was not his intention to describe them, as particular diseases, in detail, inasmuch as they would be subjects for future remarks, but merely to point out several facts of interest connected with the phenomenon just alluded to.

### PULMONARY TRANSUDATION—HÆMOPTYSIS.

The first case—one of frequent occurrence—presented several points of interest to the physician. It was that of a female—Hannah S., æt. 60—who was admitted into the Hospital on the 20th ult., for a cough of some duration, accompanied by hæmoptysis. The quantity of blood expectorated gradually increased after her entrance, although at times it yielded to the remedies employed, and a few nights since was very abundant. This was succeeded, in a few days, by copious expectoration of a bloody puriform matter, which subsequently became somewhat nummular, and at present consists almost entirely of muco-purulent matters. On examining the chest, a few days ago, the Professor detected consolidation near the summit of the left lung—one of the usual points in which tubercular deposits take place, and signs of a cavity in the inter-scapular region, near the base of the lung. The presence of this cavity was evinced by cavernous respiration, mucous rhonchi and pectoriloquy. In conjunction with these physical signs, the patient has the common vital manifestations of phthisis, as heat of surface, burning of the soles of the feet, night sweats, &c.

[The Professor here exhibited to the class the expectoration of the patient, which had been collected for several days. Some portions of it contained large quantities of blood, while others consisted almost wholly of puriform matter.]

By considering the anatomical constituents of the lung and their several relations, it can readily be understood that hæmoptysis may result in two modes,—from simple transudation through the coats of the vessels, or from ulceration and consequent hemorrhage. Thus, if tubercular matter be deposited in any portion of the lung so as to produce a mechanical impediment to the circulation, it can be easily comprehended that transudation or soaking out of the blood through the coats of the vessels may result, and that this will be much facilitated by an impoverished condition of that fluid, conjoined with softening of the pulmonary structures, as occurs in phthisis pulmonalis. Again, if the tubercular matter goes on to softening, and ulceration of the parts takes place, it can be conceived that the coats of the vessels may be destroyed, and, of course, hemorrhage may result.

[The mode in which these results may supervene, was illustrated by sketches on the black board.]

These cases are of frequent occurrence, and the importance of hæmoptysis as a diagnostic and prognostic sign must vary with the circumstances under which it has been produced. When it takes place in an individual whose lungs are free from all disease, it is of comparatively little consequence, being the result of simple hyperæmia, and the consequent transudation of the blood through the parietes of the vessels. But where it occurs in one of tuberculous diathesis, it is a most important phenomenon, inasmuch as it is symptomatic either of the first stage of tuberculosis, or of confirmed phthisis.

In the case of the patient now under consideration, we have an instance of the second form—that resulting from the formation of a cavity and ulceration of the vessels, and the hæmoptysis is, therefore, of serious import.

Combining this last phenomenon and the other vital manifestations with the physical signs, the diagnosis of pulmonary consumption in this case is well marked, although the individual's age, 60, is beyond that at which the disease usually makes its appearance.

The treatment of hæmoptysis, it need scarcely be said, must vary with the condition under which it occurs. In the present case it merges into that adapted for phthisis, which consists essentially in the use of general and topical revellents, the repeated application of counter-irritants to the thorax, with the use of cough mixtures and other palliatives. Although in cases of symptomatic hæmoptysis like this, little can be expected from remedial agents, we are called upon to prescribe something, and recourse is usually had to a combination of sedatives with astringents. Perhaps there is no better compound than the following:

℞. Acid Sulph. dil.
Tinct. Opii.
—— Digit. *aa.* ℥ij. M.

Fifteen drops to be taken three or four times daily. The opiate here is adapted for allaying irritation; the digitalis for moderating the activity of the circulation; and the acid may act, to a certain extent, as an astringent.

ENCEPHALIC TRANSUDATION—HEMIPLEGIA.

The Professor next directed the attention of the class to two cases of a similar character with the last, but where the effusion of blood was into the brain.

In these cases, there generally is precedent softening of the cerebral neurine, whereby the vessels receive less support than they ordinarily do, or the coats of the vessels are themselves diseased, or there is a combination of these pathological conditions. Such being the state of the parts, it is very obvious, that transudation of blood must take place readily under favourable circumstances, as in hyperæmia of the brain, or where impediment exists to the encephalic circulation.

One of the patients, a man, was attacked whilst asleep; for his statement is, that on awaking, prickling sensations were felt by him in various parts of the limbs; and there was a diminution of motive power on one side of the body. His locomotion is, even now, not very good, the gait being tottering. In the second patient, a female, there is great loss of power over the extremities of one side, especially of the left arm and hand. She experiences great difficulty in rising from a chair, or walking, and her face is distorted. In her case, there was more marked than in the last, what is commonly termed a "paralytic stroke." She suddenly fell from her seat, and though sensibility was retained she lost all power of motion in the affected side. A striking phenomenon occurs in all these cases; the effusion of blood always takes place on the side opposite the one which is paralysed—the cause of which, the Professor remarked, would be explained on some subsequent occasion.

In the treatment of these forms of transudation, the resources of art are very limited. There is often vascular excitement at first, which may require blood letting or other antiphlogistic means, but the subsequent condition is far from being one of excitement. Frictions, stimulating liniments, electricity, &c. are employed to the paralyzed extremities, but they are of very little efficacy, as the cause remains beyond our control: the clot of blood existing in the brain can only be removed by time: and we must rely mainly on the recuperative powers. But even after the absorption of the clot, there is not a complete restoration to health, for an apoplectic cell may remain—the evidence of previous encephalic hemorrhage—which, occupying as it does the place of some portion of the cerebral mass, must necessarily prevent the entire restoration of the lost functions.

In conclusion, the Professor exhibited some interesting

PATHOLOGICAL SPECIMENS

from a case which had fallen under his care in the wards, the history of which he gave. When he first took charge of the wards, this fall, his attention was directed to a female with enlarged abdomen, and dropsical effusion into the limbs, who was admitted for, and cured of intermittent fever, and was discharged, but for some reasons she did not leave the house. He did not examine the case at the time, but requested the resident physician to investigate the pathological cause of the phenomena. She proved to be pregnant, and, from her own statement, was at the seventh month of utero gestation. At his next visit, the lecturer found that the areolæ were not of darker colour, but there was the usual developement of the sebaceous follicles observed in pregnancy, which is considered by some as a more certain indication of pregnancy, than that derivable from the hue of the areolæ. The fact of her being pregnant was conclusively proved, however, by means of auscultation, the circulation of mother and fœtus being very perceptible. The œdema, therefore, in this case, was accounted for by the pressure of the gravid uterus on the iliac vessels, causing an effusion of the serous portions of the blood into the cellular tissue.

On the 7th inst. the patient became very much depressed in spirits, and during the evening was seized with convulsions, which, from their violence, it was feared, would produce premature labour. Examination was made if any indications of uterine contractions were present, but the neck of the uterus was found not yet obliterated, and consequently she was not transferred to the obstetrical wards. A mercurial cathartic was administered, the tongue being dry and furred. The patient remained obstinately mute, refusing to speak with any one. On the evening of the 10th, a second spasm occurred, accompanied with pains, referable to the uterus. The resident physician in charge of the ward found her on his visit in a profuse perspiration, with a rapid and full pulse—inordinate action of heart, cold extremities, left leg very œdematous, incontinence of urine; veins of the head very much congested, and the countenance distorted. She appeared unwilling to speak, but was perfectly conscious. A second examination, *per vaginam*, discovered no alteration in the condition of the uterus. The attending accoucheur was now requested to visit her, who detected slight uterine contractions, which produced, however, no dilatation of the os uteri. He ordered twelve cups to the back of the neck, to be repeated if necessary; neutral mixture every two hours, and a blister to each ankle.

The patient continued in this state of apparent hyperæmia of the brain; and was very restless. She became gradually worse from this time, and died on the 12th instant. On the day of her dissolution the abdomen was very tympanitic.

On examination, the brain exhibited an injected appearance, particularly on the surface of the hemispheres. The medullary matter on a level with the ventricles was in a state of ramollissement or softening. The other parts of the cerebrum were of the proper consistence. There was an invagination of a portion of the intestine, of four inches in length, but the other parts of the tube appeared healthy. In regard to invagination of the intestines, Professor

Dunglison remarked, that a difficulty might exist in determining whether it was morbid, or had taken place immediately preceding dissolution, owing to the irregular peristaltic action apt to occur under such circumstances. Invaginations are often met with in children, produced in the latter manner. The distinction between these—what may be called—*healthy* and the *morbid* invaginations, he observed, is easily made. If gentle traction be exerted on the intestine, and if it be not morbid, the invagination is readily withdrawn; but if morbid, in consequence of the effusion of lymph between the two peritoneal surfaces in contact, the parts become united. In this case, there was no morbid state of parts, as the invaginated portion was withdrawn with great facility, and consequently it could not be considered as having produced death. Moreover, with the exception of the tympanitis there was no evidence, during life, of intestinal lesion. The true cause of death was doubtless seated in the brain.

The placenta and cord attached to the uterus were exhibited to the class, but no morbid appearance was presented by them. The lecturer remarked, that he embraced the opportunity to exhibit them as physiological specimens.

